# Profiling of Differentially Expressed Genes Using Suppression Subtractive Hybridization in an Equine Model of Chronic Asthma

**DOI:** 10.1371/journal.pone.0029440

**Published:** 2012-01-03

**Authors:** Jean-Pierre Lavoie, Josiane Lefebvre-Lavoie, Mathilde Leclere, Anouk Lavoie-Lamoureux, Annie Chamberland, Catherine Laprise, Jacques Lussier

**Affiliations:** 1 Department of Clinical Sciences, Faculty of Veterinary Medicine, Université de Montréal, Saint-Hyacinthe, Quebec, Canada; 2 Département des Sciences Fondamentales, Université du Québec à Chicoutimi, Saguenay, Quebec, Canada; 3 Department of Biomedicine, Faculty of Veterinary Medicine, Université de Montréal, Saint-Hyacinthe, Quebec, Canada; Kyushu Institute of Technology, Japan

## Abstract

**Background:**

Gene expression analyses are used to investigate signaling pathways involved in diseases. In asthma, they have been primarily derived from the analysis of bronchial biopsies harvested from mild to moderate asthmatic subjects and controls. Due to ethical considerations, there is currently limited information on the transcriptome profile of the peripheral lung tissues in asthma.

**Objective:**

To identify genes contributing to chronic inflammation and remodeling in the peripheral lung tissue of horses with heaves, a naturally occurring asthma-like condition.

**Methods:**

Eleven adult horses (6 heaves-affected and 5 controls) were studied while horses with heaves were in clinical remission (Pasture), and during disease exacerbation induced by a 30-day natural antigen challenge during stabling (Challenge). Large peripheral lung biopsies were obtained by thoracoscopy at both time points. Using suppression subtractive hybridization (SSH), lung cDNAs of controls (Pasture and Challenge) and asymptomatic heaves-affected horses (Pasture) were subtracted from cDNAs of horses with heaves in clinical exacerbation (Challenge). The differential expression of selected genes of interest was confirmed using quantitative PCR assay.

**Results:**

Horses with heaves, but not controls, developed airway obstruction when challenged. Nine hundred and fifty cDNA clones isolated from the subtracted library were screened by dot blot array and 224 of those showing the most marked expression differences were sequenced. The gene expression pattern was confirmed by quantitative PCR in 15 of 22 selected genes. Novel genes and genes with an already defined function in asthma were identified in the subtracted cDNA library. Genes of particular interest associated with asthmatic airway inflammation and remodeling included those related to PPP3CB/NFAT, RhoA, and LTB4/GPR44 signaling pathways.

**Conclusions:**

Pathways representing new possible targets for anti-inflammatory and anti-remodeling therapies for asthma were identified. The findings of genes previously associated with asthma validate this equine model for gene expression studies.

## Introduction

Inflammation and remodeling of the airway wall are characteristic features of asthma. The term “airway remodeling” in bronchial asthma is used to describe the structural changes that occur in conjunction with, or because of, chronic inflammation. A consequence of asthmatic airway remodeling is incompletely reversible, or even irreversible airway obstruction, bronchial hyperresponsiveness, and an accelerated decline in lung function [1]. Remodeling processes in asthma result from highly complex, and poorly defined interactions between inflammatory and resident structural cells [2]. Therefore, the identification of the molecular pathways involved in the crosstalk between these cells is a prerequisite for the development of novel therapy to control airway remodeling.

Expression profile studies allow the discovery of transcripts correlated to disease phenotype and to generate hypotheses regarding genes and pathways underlying these phenotypic changes. Gene expression studies using human lung tissues have been primarily derived from the analysis of bronchial biopsies harvested from mild to moderate asthmatic subjects and controls [3]. These studies have identified candidate genes and pathways related to asthma pathogenesis. There is however limited information on the transcriptome profile of the peripheral lung tissues where remodeling predominantly occurs in non-fatal asthma [4], [5]. Using rodent models of asthma, microarrays analyses of whole lung tissues have been used to reveal the complex signaling pathways associated with the initiation of the asthmatic response. However, mice have important differences in the anatomy of the lungs compared to humans, including the relative paucity of airway smooth muscle [6]. Furthermore, sensitization to multiple antigens and recurrent challenges over many years do not occur, thus making the immune response and the crosstalk between structural cells potentially less complex than in people.

Studies of comparative pulmonary morphology show that the horse's lung closely resembles the human lung [7], [8] and their lifespan (30–35 years) is closer to human than small rodents. Also, 10 to 20% of horses develop a condition called heaves that shares many features of “extrinsic” human asthma, including lower airway inflammation, reversible airflow obstruction, and bronchial hyperresponsiveness [9], [10], [11]. Heaves develop spontaneously in susceptible horses and, similarly to asthma, is associated with increased airway smooth muscle mass, goblet cell hyperplasia, and epithelial detachment and regeneration [12], [13], [14], [15]. The horses size and temperament also allow for multiple sampling from the same animal to compare gene expression of the lung tissue under conditions of disease exacerbation and remission. Thus, equine heaves is an appealing model to study the complex inflammation-induced remodeling processes present in chronic asthma.

Suppression subtractive hybridization technique (SSH) is a highly sensitive PCR-based cDNA subtraction method [16] used to identify differentially expressed genes, including genes of relatively low abundances. It selectively amplifies differentially expressed cDNA fragments while suppressing nontarget cDNA amplification. SSH provides an approximately 1000-fold enrichment of low copy number genes related to defined phenotypes [17]. Compared to microarray analysis, SSH is more sensitive, sequence independent and yields relatively few false positive [18]. The goal of this study was to document the transcriptome associated with chronic asthmatic inflammation and tissue remodeling. We use SSH to subtract the lung transcriptome obtained from heaves-affected horses during clinical remission as well as from control horses with or without antigen exposure from lung cDNAs of horses with heaves after a 30-day antigen challenge.

## Materials and Methods

### Experimental animal model, tissue collection, and RNA isolation

Eleven horses (450–550 kg) including 9 mares and 2 geldings were studied. Six horses with heaves (mean ± SD, 16.8±2.14 years of age) had a history (mean duration 6.4±2.9 years) of recurrent episodes of airway obstruction upon hay exposure, abnormal respiratory mechanic measurements, and increased neutrophils in bronchoalveolar (BAL) fluid. Control horses (n = 5, 13.8±2.39 years of age) had no history of respiratory diseases. Detailed description of the animals, their airway function and lung inflammation have been reported elsewhere [15]. All experimental procedures were performed in accordance with the Canadian Council for Animal Care guidelines and were approved by the Animal Care Committee of the Faculty of Veterinary Medicine of the Université de Montréal (06-Rech-1324).

All animals were kept together in a low antigenic environment (Pasture) for >3 months prior to the baseline measurements and were then stabled in box stalls for 30 days where they were exposed to hay and barn dust (Challenge). Large peripheral lung biopsies were obtained at baseline and after the 30-day challenge by thoracoscopy as reported previously [19]. Biopsies were snap frozen in liquid nitrogen within 3 minutes and stored for a maximum of 5 months at −80°C until RNA extraction.

Total RNA was isolated as previously described [20]. The concentration of total RNA was quantified by measuring optical density at 260 nm using a spectrophotometer (NanoDrop ND-1000, NanoDrop products, Wilmington, DE, USA). The Agilent 2100 bioanalyzer (Agilent Technologies, Wilmington, DE, USA) was used to calculate the RNA integrity number (RIN).

### Suppression subtractive hybridization

**Figure 1:**
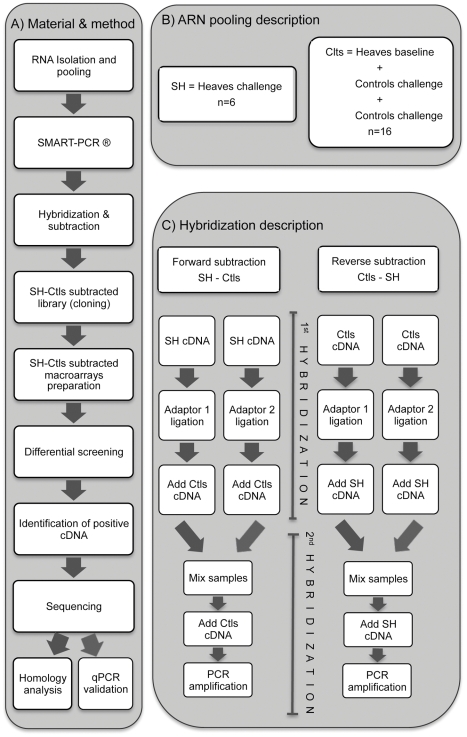
Methodology for SSH. Schematic representation of the different steps described in Material and Methods (A), the sample pooling (n corresponds to the number of samples) (B), and the different hybridization steps performed with the SSH technique (C).

SSH was used to compare gene expression in lung tissues of symptomatic heaves-affected horses during challenge (SH) versus heaves-affected horses at baseline and controls at both time points (all regrouped as “Ctls”). Identical amounts of total RNA from each horse were pooled within SH (n = 6) and Ctls (n = 16) groups to decrease inter-animal variation (Figure 1.B). The SSH procedures were performed as previously reported [21] and are illustrated in figure 1.A and C. In brief, double-stranded cDNA were generated using the SMART PCR cDNA Synthesis Kit for both SH and Ctls samples according to the manufacturer's instruction (user manual PT3041-1, Clontech Laboratories, Inc., Mountain View, CA, USA). One µg of total RNA from each pooled groups were reverse transcribed in a total volume of 10 ul with two primers (3′ SMART™ CDS Primer II A and SMART II™ A Oligonucleotide) and PowerScript reverse transcriptase (Clontech Laboratories, Inc., Mountain View, CA, USA), with the addition of 42 ng of T4 gene 32 protein (Roche Applied Science, Laval, QC, CA) to produce first cDNA strand. The resulting cDNA pools were diluted to 50 µl in TE buffer (10 mM Tris pH 8, 1 mM EDTA). Double-stranded cDNA were obtained via PCR amplification of 19 cycles with 5′PCR Primer II A using Advantage II DNA polymerase (Clontech Laboratories, Inc., Mountain View, CA, USA).

Subtracted forward (SH-Ctls) and reverse (Ctls-SH) reactions were generated by subtracting Ctls cDNAs from SH cDNAs and SH cDNAs from Ctls cDNAs, respectively, using the PCR-select cDNA Subtraction Kit (user manual PT1117-1, Clontech Laboratories, Inc., Mountain View, CA, USA). To perform the subtraction reactions, SH and Ctls cDNAs were digested with RsaI to obtain shorter, blunt-ended molecules suitable for adaptor ligation and optimal for subtractive hybridization.

Subtraction efficiency was evaluated by comparing the abundance of known genes in subtracted and unsubtracted cDNAs population after different cycles of PCR using Advantage II DNA polymerase (Clontech Laboratories, Inc., Mountain View, CA, USA). Equine gene specific primers were designed for two genes; one that is constitutively expressed, glyceraldehyde 3-phosphate dehydrogenase (*GAPDH*); and lipocalin 2 (*LCN2*), a gene known for its association with lung diseases [22] (Table S1). The subtraction efficiency was indicated by the difference in the number of cycles needed to generate equal amplification of the corresponding PCR product before and after subtraction for these two genes.

### Cloning of subtracted cDNAs

The purified (QIAquick PCR Purification kit, Qiagen, Toronto, ON, CA) subtracted (SH-Ctls) cDNAs were cloned into the pDrive-cloning vector (Qiagen, Toronto, ON, CA). Ligation products were then used to transform competent cells (DH5α, Invitrogen, Carlsbad, CA, USA), which were spread onto S-Gal®LB agar blend plate (Sigma-Aldrich Canada Ltd, Oakville, ON, CA) supplemented with kanamycin (40 µg/ml). Individual colonies (950) were transferred into ten 96-well plates containing LB freezing media (8.8% glycerol, 55 mM K_2_HPO_4_, 1 mM MgSO_4_, 26 mM KH_2_PO_4_, 15 mM NH_4_(SO_4_)) and incubated overnight to construct the SH-Ctls subtracted library.

### Differential hybridization screening

SH-Ctls subtracted macroarrays were established for differential screening, as previously described [21]. Briefly, PCR amplification was performed on the insert of each cDNA clone from the SH-Ctls subtracted library plates using AmpliTaq DNA Polymerase (Applied Biosystems, Foster City, CA, USA), with PCR-nested primers 1 and 2R (Clontech Laboratories, Inc., Mountain View, CA, USA) for 27 cycles. PCR products from each insert were denatured in 0.3 M NaOH colored with 5% bromophenol blue and vacuum-transferred onto nylon membranes (Hybond-N+, Amersham Pharmacia Biotech, Pointe-Claire, QC, CA) using a 96-well dot-blot apparatus and cross-linked to the membrane with UV light (150 mJ, GS Gene Linker, Bio-Rad, Mississauga, ON, CA). Positive control cDNA (*LCN2*) was also transferred on each membrane. For each SH-Ctls subtracted library plates, four identical cDNA macroarray membranes were generated.

Probes for the differential screening of macroarray membranes were obtained from unsubtracted (SH and Ctls) and subtracted (SH-Ctls and Ctls-SH) complex cDNA populations via secondary nested PCR amplification (PCR-select cDNA Subtraction Kit, Clontech Laboratories, Inc., Mountain View, CA, USA). Probes were then purified (QIAquick PCR Purification kit, Qiagen, Toronto, ON, CA), digested with AfaI, SmaI and EagI to remove adaptors and purified again (QIAquick PCR Purification kit, Qiagen, Toronto, ON, CA). One hundred nanograms of the cDNA probes were labeled by random priming with α^32^P[dCTP] (Megaprime DNA labeling System, GE Healthcare, Buckinghamshire, UK) as described previously [21]. Radio-labeled cDNA probes were purified (QIAquick Nucleotide Removal Kit, Qiagen, Toronto, ON, CA) and quantified with a liquid scintillation analyzer (Tri-Carb 2100TR, Packard BioScience Compagny, Meriden, CT, USA).

Each membrane was individually hybridized and washed as describe previously [21]. SH-Ctls macroarray membrane replicates were hybridized with identical amounts (cpm) of specific heat denatured cDNA probe (SH-Ctls, Ctls-SH, SH or Ctls). Washed membranes were exposed to a phosphor screen for 4 hours and the images were digitized (Storm 840, GE Healthcare, Buckinghamshire, UK). cDNA clones with different hybridization pattern were characterized using DNA sequencing and gene expression analysis.

### DNA sequencing and sequence analysis

The differentially expressed cDNA clones were amplified by PCR from the PCR products generated initially for the macroarrays using the Advantage II DNA polymerase (Clontech Laboratories, Inc., Mountain View, CA, USA) and the PCR-nested primers 1 and 2R. Amplicons were analyzed on 1.5% agarose gel with ethidium bromide to detect multiple PCR fragments. Single band amplicons were gel extracted (QIAquick Gel Extraction kit, Qiagen, Toronto, ON, CA) and sequenced via the dideoxy method (Big Dye Terminator 3.0, ABI Prism, Applied Biosystem, Foster City, CA, USA) by Génome Québec using the PCR-nested primers 1 or 2R. Sequencing reactions were analyzed with an ABI Prism 310 sequencer (Applied Biosystem, Foster City, CA, USA). Nucleic acid sequences with at least 100 bp were aligned against GenBank database (NR and EST) using the Basic Local Alignment Search Tool (BLAST). The maximum expected (E) value accepted to be considered homologous was e^−30^. Every match with higher E value score was aligned against the horse genome at the UCSC Genome Browser database using BLAT (BLAST-like alignment tool; http://genome.ucsc.edu/). Sequences were classified into two groups: I) genes with known sequence and function and II) genes with characterized sequence but unknown function. The genes from the 1^st^ group were further classified through biological function categories using functional mapping tools (GeneOntology, http://www.geneontology.org/) and compared to available literature related to human asthma and other animal models. This classification allowed us to identify biological pathways, gene families or biological functions likely to be relevant to airway remodeling and, thus, to select candidate biomarkers possibly associated with asthma.

### Validation step: Gene expression analysis

Quantitative PCR (qPCR) was used to validate the differential gene expression of 22 positive cDNA clones from the SH-Ctls library. First strand cDNA were generated using the SMART PCR cDNA Synthesis Kit (Clontech Laboratories, Inc., Mountain View, CA, USA) and the SuperScript III Reverse Transcriptase (Invitrogen, Carlsbad, CA, USA) for each individual horse at baseline and after challenge as described above. When possible, equine gene-specific primers (Table S1) were designed to span at least two intron-exon boundaries for the discrimination of contaminant genomic DNA. The absence of nonspecific products was confirmed by the analysis of the melting point curves and by electrophoresis in 1.5% agarose gels. All concentrations of target gene cDNA were calculated relatively to their respective standard curves. One microliter of cDNA template was added to the Quantitec SYBR®Green PCR Kit master mix (Qiagen, Toronto, ON, CA). qPCR reactions were performed in a volume of 20 µl using Rotor-Gene RG-3000 (Corbett Research, Sydney, AS) and qPCR conditions were similar for all primer sets (0.5 µM, final concentration): denaturation 95°C for 10 min, cycling 95°C for 15 sec, 55°C for 25 sec and 72°C for 25 sec for a maximum of 40 cycles. Each reaction was run in duplicate with the appropriate negative control. Reference gene expression was evaluated using different analysis softwares: NormFinder [23], GeNorm^PLUS^ [24] and Rest 2009 [25].

### Statistical analysis

Data are presented as mean ± SD. Differences between groups were compared at each time point using Wilcoxon tests and differences within groups were evaluated using Mann-Whitney test. Unilateral tests were used to compare values with those of asthmatic horses after 30 days of antigen challenge because SSH technique predicted the direction of the effect. Bilateral tests were used for all other analyses. P<0.05 was considered significant.

## Results

### Experimental animal model

All horses had normal lung function prior to challenge, while only horses with heaves developed clinical signs of airway obstruction and persistent airway inflammation after antigen challenge (see [15] for detailed description of lung function and BAL fluid cytology in these animals).

### Identification of differentially expressed genes

**Figure 2:**
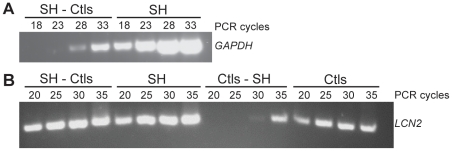
Evaluation of subtraction efficiency. A: Reduction of *GAPDH* cDNA following subtraction in the SH-Ctls sample. PCR was performed on SH-Ctls subtracted and SH unsubtracted samples. *GAPDH* PCR products (760 pb) were detectable 10 cycles earlier in the unsubtracted sample (18 cycles) than in the subtracted sample (28 cycles). B: Enrichment of *LCN2* cDNA following subtraction in the SH-Ctls sample. PCR was performed on SH-Ctls and Ctls-SH subtracted samples as well as SH and Ctls unsubtracted samples. *LCN2* PCR products (210 pb) were detected after 20 cycles for both SH unsubtracted and SH-Ctls subtracted samples, the difference in the intensity of the 2 bands indicate the enrichment compare to Ctls unsubtracted and Ctls-SH subtracted samples.

The quality of the RNA samples was confirmed by high RIN values (8.47±0.58 SD) and electropherogram analysis. Subtraction efficiency was evaluated using standard PCR for two genes; *GAPDH* and *LCN2*. *GAPDH* PCR products were detectable after only 18 cycles in the SH unsubtracted sample, whereas 10 more cycles were required to detect the PCR fragment in the SH-Ctls subtracted sample (Figure 2.A), a 40 fold reduction. *LCN2* PCR products were detectable after 20 cycles in both subtracted SH-Ctls and unsubtracted SH samples, but the difference in intensity between the two signals indicates enrichment (Figure 2.B). Conversely, the *LCN2* PCR products were detected after 20 cycles in the unsubtracted Ctls sample and after 10 more cycles (40 fold increase) in the reverse subtracted Ctls-SH sample.

**Figure 3:**
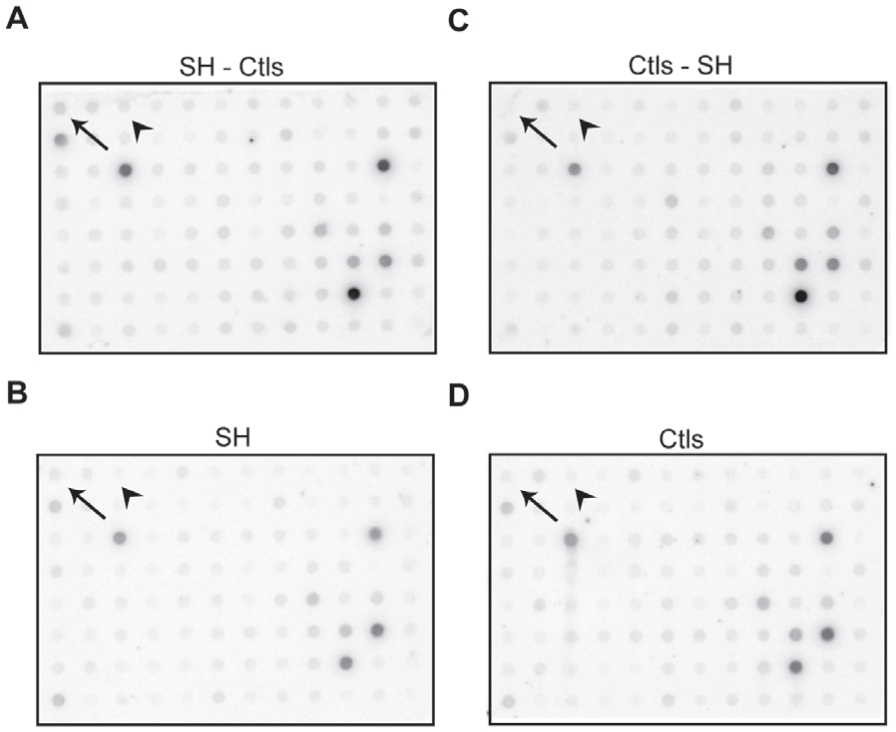
Differential hybridization screening. Representative differential screening results of macroarrays of the SH-Ctls library. Four identical membranes were dot-blotted with PCR products obtained by SSH. The membranes were then hybridized with four different probes: SH-Ctls subtracted cDNAs (A), SH unsubtracted cDNAs (B), Ctls-SH subtracted cDNAs (C) and Ctls unsubtracted cDNAs. The arrow in the top left corner indicates the positive control (*LCN2*). The arrow head indicates an example of differentially expressed genes in SH compare with Ctls.

Differential hybridization screening was performed using macroarrays in order to isolate genes implicated in heaves exacerbation from the 950 randomly selected clones. Differentially expressed cDNA clones were identified based on the hybridization signal intensities observed between the four membranes. The positive clones had 1) a stronger hybridization signal with the SH-Ctls probe than with SH probe, 2) a weaker hybridization signal with the Ctls-SH probe than with the Ctls probe, and 3) a stronger hybridization signal with the SH-Ctls probe than with the Ctls-SH probe. Representative differential screening results are illustrated in Figure 3. Of the 950 cDNA clones screened, 294 were identified as strongly expressed and were analyzed on agarose gel. Sequencing performed on single band PCR amplicons generated a total of 224 clones with adequate sequencing results for BLAST analysis and Genbank deposition (accession numbers from GH613643 to GH613840).

The first group contained 167 sequences with known function, 20 of which were redundant. These sequences were further categorized based on their biological pathways (Table S2, 147 sequences). The second group contained 57 sequences previously characterized, but with unknown functions; 9 of these sequences were redundant (Table S2, 48 sequences). In group I, there were 14 genes related to regulatory proteins, 14 to immune signaling molecules, 13 to intracellular signaling component pathways, 10 to immune response, 5 to cell growth and proliferation, 5 to free radical metabolism (Table S2, 61 genes). There were 86 additional genes with known cell function (6 to transmembrane proteins, 10 to structural proteins, 4 to extracellular proteins, 1 to complement components, 4 to gene transcription, 2 to cell adhesion molecules, 2 to metal ion binding, 24 to DNA/RNA associated proteins, 11 to transport proteins, 13 to metabolic enzymes, 4 to proteolytic enzymes and 5 to protein binding).

### Gene expression analysis

**Figure 4:**
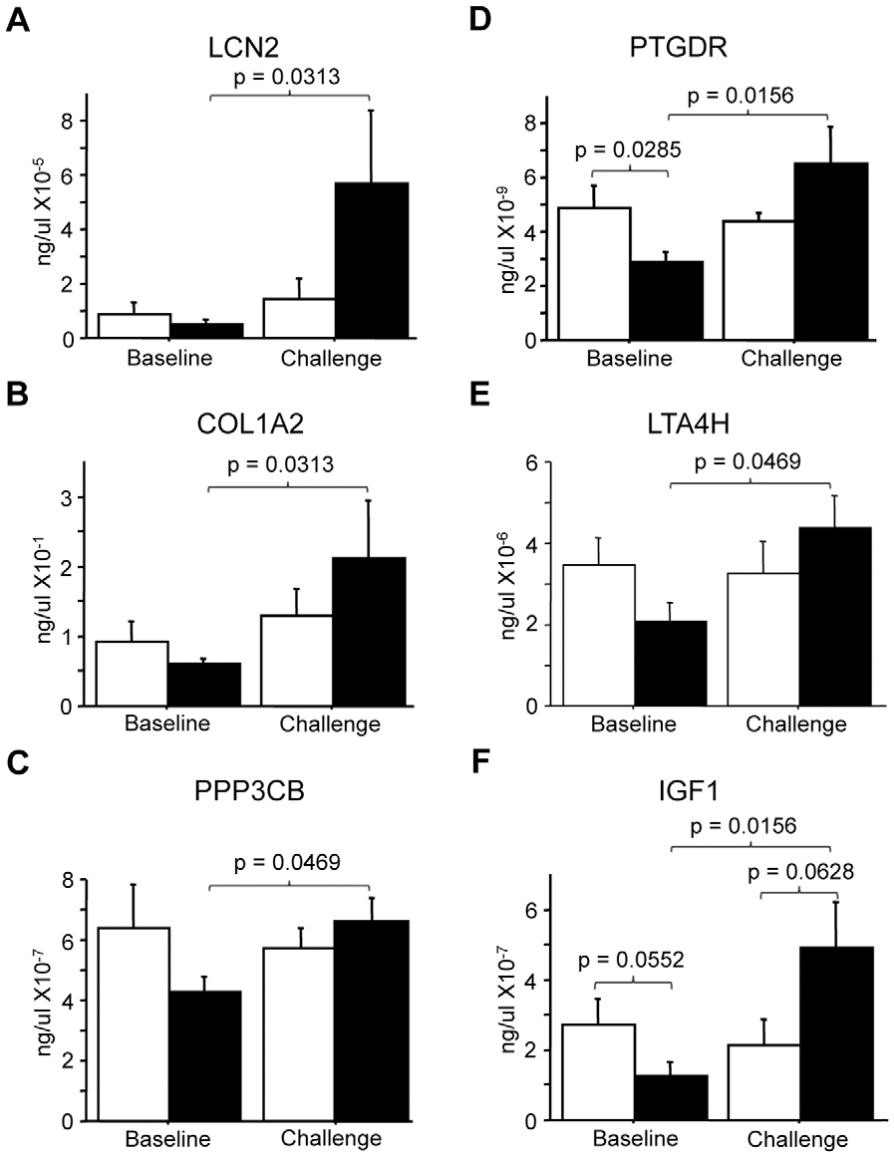
Gene expression analysis. Analysis of mRNA expression using qPCR of six genes found up-regulated with SSH. *LCN2* (A), *COL1A2* (B), *PP3CB* (C), *PTGDR* (D), *LTA4H* (E) and *IGF1* (F) were studied in six horses with heaves (black bars) and six control horses (white bars). When compared to baseline, the six genes were significantly increased in heaves-affected horses after the allergen challenge (p<0.05). *PTDGR* was also increased in control horses when compared to heaves-affected horses at baseline.

To confirm the differential gene expression pattern in heaves-affected horses, mRNA expression was compared by qPCR in individual heaves and control horses before and after challenge. The 22 genes selected for validation were chosen because they had previously been associated with asthma, or because of their possible contribution to airway inflammation and remodeling. They included *LCN2*, collagen type I alpha 2 (*COL1A2*), collagen type III alpha 1 (*COL3A1*), protein phosphatase 3 catalytic subunit beta (*PPP3CB*), glypican 4 (*GPC4*), versican (*VCAN*), chemokine (C-C motif) ligand 5 (*CCL5*), decorin (*DCN*), major histocompatibility complex class II invariant chain CD74 molecule (*CD74*), dedicator of cytokinesis 1 (*DOCK1*), fucosidase alpha-L-1 (*FUCA1*), mitochondrial translational release factor 1-like (*MTRF1L*), NHL repeat containing 2 (*NHLRC2*), prostaglandin D2 receptor (*PTGDR*), leukotriene A-4 hydrolase (*LTA4H*), endothelin receptor type A (*EDNRA*), chemokin binding protein 2 (*CCBP2*), insulin-like growth factor I (*IGF1*), gamma actin (*ACTG1*), vimentin (*VIM*), TRPC4 associated protein (*TRPC4AP*) and Rho GTPase activating protein 25 (*ARHGAP25*). *LCN2*, *COL1A2*, *PPP3CB*, *VCAN*, *DCN*, *CD74*, *NHLRC2*, *IGF1*, *FUCA1* and *LTA4H* mRNA were significantly increased in heaves-affected horses after the challenge compared to baseline, in contrast to the mRNA expression in controls, which remained stable during the study. Similarly, *PTGDR* and *MTRF1L* also showed a significant increase in heaves-affected horses after challenge compared to baseline, but in addition, the baseline mRNA expression was significantly higher in the control group compared to the heaves-affected group. The expression of *ARHGAP25* and *ACTG1* mRNA were significantly decreased after challenge compared to baseline in control horses only. *ARHGAP25*, *EDNRA* and *ACTG1* were also significantly different between groups at baseline, control horses having higher mRNA expression. There were no significant differences between groups and between time points in each group in *COL3A1*, *GPC4*, *CCL5*, *DOCK1*, *CCBP2*, *TRPC4AP* and *VIM*. Figure 4 represents the mRNA expression using qPCR for six of the genes found to be upregulated with SSH.

Quantification for genes of interest is expressed as absolute concentration because all reference genes tested showed significant (p<0.05) increase in the lung tissue of heaves-affected horses after the antigen challenge compared to baseline. The reference genes tested were *GAPDH*, ubiquitin C (*UBC*), b-glucuronidase (*GUSB*), ß2-microglobulin (*B2M*), peptidylprolyl isomerase A (*PPIA*), large ribosomal protein P0 (*RPLP0*), and ribosomal protein S9 (*RPS9*). Reference gene analysis using dedicated softwares further confirmed that the stability of these genes was highly dependent on horses' clinical status, precluding their use for normalization. In view of these results, total RNA and reverse transcribe (RT) reactions were quantified using a spectrophotometer (NanoDrop ND-1000, NanoDrop products, Wilmington, DE, USA) and used to normalize cDNA quantity in the PCR reactions [26]. Lastly, to ensure that unidentified biases were not introduced during sample analysis, the upregulation of *GAPDH*, *PPIA*, *LTA4H*, *PPP3CB* and *LCN2* in horses with heaves after antigen stimulation was confirmed (data not shown) using samples of lung tissues from the same animals but archived using a different technique of preservation (RNA later, Ambion, Austin, TX, USA), RNA extraction (TRIzol® Invitrogen, Carlsbad, CA, USA) and a different enzyme for RT reactions (AMV, Roche Diagnostics Corp, Laval, QC, CA).

## Discussion

Large-scale expression studies have highlighted complex interactions occurring between genes and environment in the central asthmatic airways [27] and allowed the discovery of novel pathways implicated in the disease inflammatory and remodeling processes. In the present study, we generated a reciprocal cDNA library representing mRNA specific to the peripheral lung of asthmatic horses during exacerbation. This led to the identification of 195 genes, 75.4% of which corresponded to genes with known sequences and functions and 24.6% to uncharacterized cDNAs. Quantitative PCR confirmed the differential gene expression pattern in 15 of the 22 genes evaluated. The functions of many of these genes were related to inflammation, remodeling, and smooth muscle biology, possibly representing new therapeutic targets for asthma.

### Genes associated with remodeling and smooth muscle contraction

Inflammation and repair of injured lung tissues in asthma results in an increased thickness of the airway wall leading to reduced baseline airway caliber and exaggerated airway narrowing, phenomena that are accentuated in allergen-induced bronchospasm [28]. Of particular importance to asthma is the increased airway smooth muscle (ASM) mass observed in human subjects (reviewed by [29]) and in equine heaves [14], [15]. Not only are ASM cells increased in number or size and contribute to the bronchospasm, but they show, at least *in vitro*, some phenotype plasticity in response to allergen challenge, including de-differentiation to a more synthetic type capable of producing an extracellular matrix (ECM), various cytokines, and growth factors (reviewed by [30]). However, the molecular pathways responsible for these changes are poorly defined. Herein, we identified at least 13 genes that have been linked to smooth muscle biology. Four genes (*ARHGAP25*, phosphatidylinositol transfer protein alpha (*PITPNA*), phosphatidylinositol-specific phospholipase C X domain containing 3 (*PLCXD3*), and pyruvate dehydrogenase kinase isozyme 1 (*PDK1*)) identified in the lung tissues of heaves-affected horses in exacerbation are involved in the modulation of the RhoA pathway [31]. This pathway is necessary for the activation of factor serum response factor (*SRF*) by myocardin [32], one of its coactivators, which leads to the expression of contractile protein genes expression [33]. Interestingly, RhoA/Rho kinase is required for ASM contraction induced by endothelin-1 (*EDN1*) [34] and is upregulated by interleukin-4 (*IL-4*), a Th2 cytokine expressed in the airways of asthmatic patients [35]. Our results are thus in agreement and extend those of *in vitro* and animal studies, and further support the proposal of Rho kinase inhibitors as new targets for the treatment of airway bronchoconstriction and remodeling seen in asthma (reviewed by [36]). Conversely, it is the mitogen-activated protein kinase (*MAPK*) signaling pathway that promotes smooth muscle proliferation by modulating *SRF*-transcriptional activities via the activation *Elk-1* [32], [33]. *MAPK1* identified in our SSH activates *Elk-1* [37], suggesting that both ASM proliferation and differentiation may coexist in the asthmatic lungs.

*PPP3CB* (also known as calcineurin) and *IGF1* identified by SSH share common signaling pathways also possibly contributing to smooth muscle phenotype switching and ECM remodeling in asthma [38], [39], [40], [41], [42]. The identification of *PPP3CB*, and one of its inhibitors, calcineurin homologous protein (*CHP*) [43], is of particular interest as *PPP3CB*/*NFAT* signaling is implicated in a wide range of biological responses relevant to asthma including lymphocyte activation, as well as neuronal and muscle development [43], [44], [45]. While not yet investigated in lung tissues to our knowledge, *PPP3CB* activation results in muscle hypertrophy in response to increase workload in both the urinary bladder and in the heart [46], [47], [48]. Furthermore, alterations of the expression of the fast and slow myosin heavy chain isoforms in the obstructed bladder is *PPP3CB*-dependent [48]. Thus the *PPP3CB* pathway may participate in the increased ASM mass and the myosin heavy chain isoform switching observed in the asthmatic airways [49]. The expression of *EDN1*, a potent spasmogen for the bronchus, is increased in asthma [50], and single nucleotide polymorphisms (SNPs) have been associated with susceptibility to this disease [51]. One of its receptor, *EDNRA*, identified in our SSH, has previously been found to be upregulated in this animal model [52]. Interestingly, the *PPP3CB*/*NFAT* pathway discussed above has been shown to be required for at least some of the effects of *EDN1* in cardiac myocytes [39], [53], and thus, are further support for their possible modulation of ASM remodeling.

*IGF1* plays a vital role in embryonic development and promotes the anabolism and the repair of various tissues in adults [54]. There are several evidences suggesting that *IGF1* may also contribute to asthma. *IGF1* is produced by human bronchial epithelial cells in response to *IL-17F* [55], a cytokine implicated in asthma. It was shown to induce the expression of alpha-smooth muscle actin and type-I collagen by human fetal lung fibroblasts [56], and to promote visceral myocyte differentiation into a contractile phenotype via the *PPP3CB*/*NFAT* pathway [57], [58]. The increased expression of *IGF1* in the peripheral lung tissue of horses with heaves during exacerbation is thus of interest to asthma, especially in the light that *IGF1* neutralizing antibody inhibits airway obstruction and inflammation, while preventing airway wall thickening in a mouse model of asthma [59].

There is also alteration of various components of the ECM in the asthmatic airways. These changes vary depending on size of airways, and it has been shown that an increase in the degree of subepithelial fibrosis correlates with an increase in the severity of asthma [60]. Not surprisingly, gene expression of ECM molecules including collagens (*COL1A2*, *COL3A1*), and proteoglycans (*DCN*, *GPC4*, *VCAN*) were identified by SSH. The expression of collagen, type I, and type III led us to investigate the total collagen content in the airways of these horses which revealed to be increased (unpublished data). The increased expression of the intermediate filaments *VIM* may also be relevant as it is required for epithelial to mesenchymal transition, a phenomenon where epithelial cell properties change from non-migrational to a fibroblastic and migrational-mesenchymal cell type, which has been proposed to be contributing to the increased ASM mass observed in asthma [61].

### Genes associated with inflammation

Pulmonary inflammation is a characteristic finding in asthma and anti-inflammatory drugs are central for its control. Seven genes associated with leukotriene (*LT*)B4 metabolism or prostaglandin (*PG*)D2 activity were identified as being overexpressed in the lungs of horses with heaves during exacerbation. Those included LTA4 hydrolase which metabolizes *LTA4* in *LTB4*, PGF synthase that reduces *PGD2* and *PGH2* to *PGF2*, *PTGDR* (also named *DP1*), a *PGD2* receptor involved in the regulation of Th2-type driven inflammation [62], and *CNOT7* (CCR4-NOT transcription complex), a repressor of the retinoid X beta receptor (*Rxrb*) [63], which forms a heterodimeric complex with the nuclear receptors *PPARs* (peroxisome proliferator-activated receptor). *LTB4* is an arachidonic acid metabolite synthesized by various cell types when activated by inflammatory stimuli. *LTB4* was first described as a potent chemoattractant and activator of neutrophils, the predominant airway cell population present in heaves, and in some asthmatic patients [64]. It is now recognized that *LTB4* also exerts these effects on other cell types involved in airway inflammation [65] and it has also been suggested that it is implicated in T cell trafficking and asthmatic inflammation [66]. Further support for a role of *LTB4* in asthma is its increase in exhaled breath condensate of affected patients [67], and the attenuation of allergic airway inflammation and hyperresponsiveness by *LTA4H* inhibition [68]. However, the effects of *LTA4H* are complex, as it can also limit tissue damage-induced neutrophilia through its aminopeptidase activity which degrades proline-glycine-proline (PGP), a collagen breakdown product possessing potent neutrophil chemotactic activity [69]. *PGD2* is also an arachidonic acid metabolite that is released in large quantities by mast cells during anaphylaxis. Other cell types present in lung tissues such has dendritic cells, macrophages, eosinophils, Th2 cells, and endothelial cells may also produce *PGD2*, and contribute to asthmatic inflammation [66]. *PGD2* exerts its effects by activating two distinct G protein-coupled receptors, including the *PTGDR* identified by SSH. It has been recently proposed that *PTGDR* may regulate *PGD2*-directed T-cell trafficking and Th2-dependent airway inflammation [62], [66]. These results suggest that the pharmacological modulation of these lipidic mediators represent possible novel therapeutic targets for the treatment of human asthma [70].

In summary, we have identified genes and pathways relevant to the asthmatic inflammation and remodeling that were upregulated in the peripheral lung tissues of horses with heaves when antigen challenged. Genes previously associated with asthma as well as novel pathways were also identified. These genes encompass a range of biological processes with pathways related to ASM and ECM remodeling, and inflammation being notable. Our results suggest that targeting *RhoA*, *PPP3CB*, *EDN1*, and *IGF1* signaling pathways may represent appropriate targets for anti-remodeling therapies, especially for the control ASM hypertrophy, while anti-inflammatory effects may possibly be achieved by drugs modulating *LTB4* and *PGD2*.

## Supporting Information

Table S1 **Sequences of primer pairs used for PCR analysis.**
(DOCX)

Table S2 **Identification and functional classification of differentially expressed transcripts in horses with heaves during airway obstruction when compared to healthy controls and asymptomatic asthmatic horses.**
(DOCX)
