# Supplementary material for: Profiling of Differentially Expressed Genes Using Suppression Subtractive Hybridization in an Equine Model of Chronic Asthma
Source: PLoS One. 2012 Jan 3;7(1):e29440. doi: 10.1371/journal.pone.0029440 (PMC3250435; doi:10.1371/journal.pone.0029440)
Supplement: Table S1 — Sequences of primer pairs used for PCR analysis. (DOCX) [file pone.0029440.s001.docx]

**Table S1. Sequences of primer pairs used for PCR analysis.**

| **Gene Id** | **Forward sequence (5’–3’)** | **Reverse sequence (5’–3’)** | **Amplicon length** | **Intron spanning** |
| --- | --- | --- | --- | --- |
| *GAPDH*-std | caagttccatggcacagtcaagg | aaagtggtcgttgagggcaatgc | 760 | yes |
| *LCN2* | accaccacctacgagctgaagg | ctgaagaacacgatggcaaactgg | 210 | yes |
| *GAPDH* | aagtggatattgtcgccatcaat | aacttgccatgggtggaatc | 88 | yes |
| *COL1A2*-std | ctaccactgcaagaacagcattgc | ccacaacaggtgtaagagtgctg | 547 | no |
| *COL1A2* | gtcactctgcaaggctccaatgat | ccaccgatgtccaaaagtgcaatg | 185 | yes |
| *COL3A1* | ttgtgcaaaaggggacctggtt | gacgcatatttggcatggttctgg | 152 | yes |
| *PPP3CB* | aggatcggaccatagcaagtcaca | agcttttgatagcctgcctcttgc | 166 | yes |
| *GPC4* | acgtgtccaaaggcttcgacaa | ctgctcgctgaccacacttatgaa | 161 | yes |
| *VCAN* | gcgctgatccctaaaatggcaaac | ggagcacagcaatcccaaatgact | 148 | no |
| *CCL5* | ctttcgggtgacaaagacgact | accttctgcactcctgcatct | 159 | yes |
| *DCN* | aggctagctgcatcaactttggtg | agaagctgtcctacatccgcattg | 124 | yes |
| *CD74* | gccaaacctgtgagcaagattcg | tcctgtgtcgtgttcccgtactt | 119 | yes |
| *DOCK1* | agctgcaccatcagcaaagact | ttggtgttggaacgccacttca | 110 | yes |
| *FUCA1* | gaccacaaagcatcacgaaggcta | ctctggcattgttttcgcactgac | 241 | yes |
| *MTRF1L* | ccactggctcgcttagtatcgatt | atccacaccagcaccatgacagta | 104 | yes |
| *NHLRC2* | tcacctgttgcctgatcttcatgc | aggaaacttccagctcttgcca | 200 | yes |
| *PTGDR* | atgcgcaacctctacacgatg | caggtctaaacgctccaacggtaa | 209 | yes |
| *LTA4H* | tgtcaggacactccttccgtgaaa | agatgcttctccatcacgaatggc | 102 | yes |
| *EDNRA* | tgagaattgccctcagtgaacacc | atgaagagggaaccagcaaagagc | 101 | yes |
| *CCBP2* | tacctggagatcgtccatgctcaa | aatggtcccatgccctccaaaatc | 189 | no |
| *IGF1* | gtgtgtggagacaggggcttttat | acttccttctgagccttgggcata | 215 | yes |
| *ACTG1* | atcgtgcgtgacatcaaggagaag | gtgttagcatacaggtccttgcga | 269 | yes |
| *VIM* | ggcgaagcaggagtcaaatgagta | aggtcttggtattcccgaaggtga | 225 | yes |
| *TRPC4AP* | atgtccttcctcttccgcctcatt | tgttgagcaggaagccaggatact | 187 | yes |
| *ARHGAP25* | ccctgtggccagattcaaaaggat | cccctggaaaacacagcataccat | 289 | no |
| *UBB* | tagcagtttcttcgttgtccgt | tgtaatcggaaagagtgcgg | 211 | yes |
| *GUSB* | gctcatctggaactttgctgatttt | ctgacgagtgaagatcccctttt | 85 | yes |
| *B2M* | tcgggctactctccctgactg | cggcaactatactcatccacacca | 271 | yes |
| *PPIA* | tgctggacccaacacaaatggttc | gtccacagtcagcaatggtgatct | 182 | yes |
| *RPLP0* | gctgatgggcaagaacaccatgat | ggtaaacacaaagcccacattgcc | 115 | yes |
| *RPS9* | tcaaattcaccctggccaagatcc | gcgcctctccaagaaatcctctat | 191 | yes |
