# Supplementary material for: Profiling of Differentially Expressed Genes Using Suppression Subtractive Hybridization in an Equine Model of Chronic Asthma
Source: PLoS One. 2012 Jan 3;7(1):e29440. doi: 10.1371/journal.pone.0029440 (PMC3250435; doi:10.1371/journal.pone.0029440)
Supplement: Table S2 — Identification and functional classification of differentially expressed transcripts in horses with heaves during airway obstruction when compared to healthy controls and asymptomatic asthmatic horses. (DOCX) [file pone.0029440.s002.docx]

**Table S2: Identification and functional classification of differentially expressed transcripts in horses with heaves during airway obstruction when compared to healthy controls and asymptomatic asthmatic horses.**

| **Gene Identification** | | **Homology** | | |
| --- | --- | --- | --- | --- |
| **Fr** | **Gene correspondance** | **Accession number** | **Sp** | **Identity %** |
| **Sequences coding with known genes - Group I** | | | | |
| **Immune signaling molecules** | | | | |
| 1 | **ANXA1**: annexin I | X95108 | SC | 85 |
| 1 | **BANK1**: B-cell scaffold protein with ankyrin repeats 1 | XM_001108807 | MM | 81 |
| 1 | **CCBP2**: chemokine binding protein 2 | XM_001497290 | EC | 99 |
| 1 | **CCL5**: chemokine (C-C motif) ligand 5 | NM_001081863 | EC | 100 |
| 1 | **CD74**: CD74 molecule, major histocompatibility complex class II invariant chain | NM_001099770 | EC | 98 |
| 1 | **IK**: IK cytokine, down-regulator of HLA II | NM__001103349 | BT | 94 |
| 1 | **IL6ST**: interleukin 6 signal transducer | XM_517756 | PT | 77 |
| 2 | **LCN2**: lipocalin 2 | XM_001501148 | EC | 99 |
| 1 | **LCP2**: lymphocyte cytosolic protein 2 (SH2 domain containing leukocyte protein of 76kDa) | XM_003310976 | PT | 82 |
| 1 | **PTGDR**: prostaglandin D2 receptor (DP) | NM_001098034 | BT | 70 |
| 1 | **SFTPA1**: surfactant protein A1 | NM_001009728 | OA | 78 |
| 1 | **SPP1**: secreted phosphoprotein 1 | XM_001496174 | EC | 100 |
| 1 | **TXNIP**: thioredoxin interacting protein | XM_002924566 | AM | 83 |
| 1 | **SOCS5:** suppressor of cytokine signaling 5 | XM_001498720 | EC | 99 |
| **Immune response** | | | | |
| 1 | **BCAP29**: B-cell receptor-associated protein 29 | NM_001038075 | BT | 89 |
| 1 | **DOCK1**: dedicator of cytokinesis 1 | XM_001489764 | EC | 100 |
| 1 | **GBP2**: guanylate binding protein 2, interferon-inducible | XM_001494427 | EC | 100 |
| 2 | **IGHA1**: immunoglobulin heavy constant alpha 1 | AY247966 | EC | 94 |
| 2 | **IGL@:** immunoglobulin lambda locus | XM_001915391 | EC | 95 |
| 1 | **LRBA**: LPS-responsive vesicle trafficking, beach and anchor containing | XM_001501505 | EC | 100 |
| 1 | **PSMC1**: proteasome (prosome, macropain) 26S subunit, ATPase, 1 | XM_001494090 | EC | 99 |
| 1 | **SEMA3C**: sema domain, immunoglobulin domain (Ig) short basic domain secreted (semaphorin) 3C | XM_003252189 | NL | 80 |
| 1 | **SEPP1**: selenoprotein P, plasma, 1 | NM_001135605 | EC | 99 |
| 1 | **ZFAND5**: zinc finger AN1-type domain 5 | XM_001488383 | EC | 98 |
| **Intracellular signaling** | | | | |
| 1 | **CHP**: calcineurin B homologous protein | BT030715 | BT | 82 |
| 1 | **EVI2B:** ecotropic viral integration site 2B | XM_001504035 | EC | 99 |
| 1 | **GAB1**: GRB2-associated binding protein 1 | NM_001101201 | BT | 83 |
| 1 | **MAPK1**: mitogen-activated protein kinase 1 | NM_002745 | HS | 94 |
| 1 | **MLLT4:** myeloid/lymphoid or mixed-lineage leukemia (trithorax homolog, Drosophila); translocated to 4 | XM_581038 | BT | 76 |
| 1 | **PLCXD3**: phosphatidylinositol-specific phospholipase C, X domain containing 3 | NM_001005473 | HS | 89 |
| 1 | **PPP3CB**: protein phosphatase 3 (formerly 2B), catalytic subunit, beta isoform | XM_001502920 | EC | 99 |
| 1 | **PITPNA**: phosphatidylinositol transfer protein alpha | BC082976 | HS | 81 |
| 1 | **RAB18**: member RAS oncogene family | XM_001494294 | EC | 99 |
| 1 | **TRPC4AP**: transient receptor potential cation channel, subfamily C, member 4 associated protein | XM_001499199 | EC | 99 |
| 2 | **YWHAB**: tyrosine 3-monooxygenase/tryptophan 5-monooxygenase activation protein, beta polypeptide | AB169570 | MF | 83 |
| 1 | **ARPC5:** actin-related protein 2/3 complex subunit 5 | XM_001915096 | EC | 98 |
| 1 | **ZBTB8OS:** zinc finger and BTB domain containing 8 opposite strand | XM_001503767 | EC | 96 |
| **Proteolytic enzymes** | | | | |
| 2 | **GLRX**: Glutaredoxin (Thioltransferase) | XM_846466 | CF | 79 |
| 1 | **LTA4H**: Leukotriene A-4 hydrolase | XM_001494765 | EC | 100 |
| 1 | **PSMA5**: proteasome (prosome, macropain) subunit alpha type 5 | XM_001918054 | EC | 99 |
| 1 | **USP47**: ubiquitin specific peptidase 47 | XM_001501192 | EC | 99 |
| **Extracellular proteins** | | | | |
| 1 | **APOOL**: apolipoprotein O-like | NM_198450 | HS | 88 |
| 1 | **CCDC80**: coiled-coil domain containing 80 | XM_001502981 | EC | 100 |
| 2 | **COL1A2**: collagen type I alpha 2 | XM_001492939 | EC | 99 |
| 1 | **COL3A1**: collagen type III alpha 1 | XM_001917620 | EC | 99 |
| **Transmembrane proteins** | | | | |
| 1 | **EDNRA**: endothelin receptor type A | XM_862339 | CF | 80 |
| 1 | **MAL2:** mal, T-cell differentiation protein 2 | XM_001142529 | PT | 82 |
| 1 | **NIPA1**: non-imprinted in Prader-Willi/Angelman syndrome 1 | XM_523025 | PT | 81 |
| 1 | **PMP22**: peripheral myelin protein 22 | XM_002800299 | MM | 86 |
| 1 | **TMEM19**: transmembrane protein 19 | NM_018279 | HS | 73 |
| 1 | **LAPTM5:** lysosomal protein transmembrane 5 | XM_001503920 | EC | 78 |
| **Complement components** | | | | |
| 1 | **C4BPA**: complement component 4 binding protein alpha | XM_001492532 | EC | 99 |
| **Regulatory proteins** | | | | |
| 1 | **ARHGAP25**: Rho GTPase activating protein 25 | NM_001046392 | BT | 75 |
| 1 | **CCNB1**: Cyclin B1 | XM_001491280 | EC | 100 |
| 1 | **FTH1**: ferritin heavy polypeptide 1 | NM_001100413 | EC | 99 |
| 1 | **GM2A**: GM2 ganglioside activator | NM_001205547 | BT | 81 |
| 1 | **GPBP1**: GC-rich promoter binding protein 1 | XM_001495419 | EC | 100 |
| 1 | **HSPC159**: galectin-related protein | NM_001205831 | BT | 90 |
| 1 | **MOBKL1B**: MOB1, Mps One Binder kinase activator-like 1B (yeast) | NM_001132280 | PA | 77 |
| 1 | **NPHP3**: nephronophthisis 3 (adolescent) | AB258811 | EC | 82 |
| 1 | **NR2F2**: nuclear receptor subfamily 2 group F member 2 | NM_174402 | BT | 97 |
| 1 | **PRDX6:** peroxiredoxin 6 | XM_001496832 | EC | 99 |
| 1 | **SLITRK4:** SLIT and NTRK-like family member 4 | XM_001490209 | EC | 100 |
| 1 | **SRI**: sorcin | XM_001492148 | EC | 99 |
| 2 | **TPT1**: tumor protein, translationally-controlled 1 | XM_001491304 | EC | 99 |
| 1 | **YWHAZ**: tyrosine 3/tryptophan 5 -monooxygenase activation protein zeta polypeptide | XM_001492988 | EC | 99 |
| **Cell growth and proliferation** | | | | |
| 1 | **CUL2**: cullin 2 | XM_851747 | CF | 95 |
| 1 | **DONSON**: downstream neighbor of SON | XM_001494421 | EC | 99 |
| 1 | **GPC4**: glypican 4 | XM_001097252 | MM | 80 |
| 1 | **IGF1**: insulin-like growth factor I | XM_001156521 | PT | 75 |
| 1 | **USP16**: ubiquitin specific peptidase 16 | NM_001032410 | HS | 93 |
| **Cell adhesion molecules** | | | | |
| 1 | **GPM6A**: glycoprotein M6A | XM_001493131 | EC | 99 |
| 1 | **VCAN**: versican | NG_012682 | HS | 94 |
| **Structural proteins** | | | | |
| 1 | **ACTB**: beta-actin | AB289605 | TT | 90 |
| 1 | **EPB41L2**: erythrocyte membrane protein band 4.1-like 2 | XM_002690221 | BT | 93 |
| 3 | **FGA**: fibrinogen alpha chain | XM_001915158 | EC | 100 |
| 2 | **ACTG1**: gamma actin 1 | XM_001488883 | EC | 99 |
| 1 | **RPL10**: ribosomal protein L10 | XM_001915513 | EC | 100 |
| 1 | **OPA1**: optic atrophy 1 | XM_001500454 | EC | 99 |
| 1 | **DCN**: decorin | NM_001081925 | EC | 99 |
| 2 | **PLS3:** plastin 3 | XM_001488277 | EC | 99 |
| 1 | **SMC5**: structural maintenance of chromosomes 5 | XM_001489222 | EC | 99 |
| 1 | **VIM:** Vimentin | XM_001916285 | EC | 99 |
| **Transport proteins** | | | | |
| 1 | **ABCE1**: ATP-binding cassette sub-family E (OABP) member 1 | DQ148409 | HS | 72 |
| 1 | **ATP6V1D**: ATPase, H+ transporting, lysosomal 34kDa, V1 subunit D | XM_001499730 | EC | 98 |
| 1 | **ATP11B**: ATPase class VI type 11B | XM_001496792 | EC | 100 |
| 1 | **EXOC1**: Exocyst complex component 1 | XM_001492690 | EC | 99 |
| 1 | **FTL**: ferritin light polypeptide | XM_001251063 | BT | 79 |
| 1 | **KCTD20**: potassium channel tetramerisation domain containing 20 | NM_001101093 | BT | 78 |
| 1 | **IPO5**: importin 5 | XM_001492682 | EC | 97 |
| 1 | **SEC61G**: Sec61 gamma subunit | NM_001040586 | BT | 94 |
| 1 | **SLCO3A1**: solute carrier organic anion transporter family, member 3A1 | XM_001916720 | EC | 99 |
| 1 | **UACA**: uveal autoantigen with coiled-coil domains and ankyrin repeats | XM_002804842 | MM | 89 |
| 3 | **USO1**: USO1 vesicle docking protein homolog | XM_001490004 | EC | 99 |
| **Metabolic enzymes** | | | | |
| 1 | **ACSL5**: acyl-CoA synthetase long-chain family member 5 | NM_203380 | HS | 91 |
| 1 | **ACSS2**: acyl-CoA synthetase short-chain family member 2 | NM_001076552 | HS | 82 |
| 1 | **PGFS**: prostaglandin F synthase | NM_001081895 | EC | 99 |
| 1 | **ALDH1A1**: aldehyde dehydrogenase 1 family, member A1 | XM_003267414 | NL | 84 |
| 1 | **EXT2**: exostosin 2 | NM_207122 | HS | 76 |
| 1 | **FUCA1**: fucosidase alpha-L- 1, tissue | X92448 | CF | 79 |
| 1 | **GLB1**: galactosidase beta 1 | XM_001490147 | EC | 99 |
| 2 | **LDHA**: lactate dehydrogenase-A | GU808337 | EC | 99 |
| 1 | **METTL7A**: methyltransferase like 7A | NM_001035439 | BT | 77 |
| 1 | **PDK1**: pyruvate dehydrogenase kinase isozyme 1 | XM_534032 | CF | 80 |
| 1 | **PIGB**: Phosphatidylinositol glycan anchor biosynthesis class B | XM_001501000 | EC | 99 |
| 1 | **PPT1**: palmitoyl-protein thioesterase 1 | NM_001010944 | CF | 85 |
| 1 | **BPNT1:** 3(2), 5-biphosphate nucleotidase 1 | XM_001488034 | EC | 100 |
| **Protein binding** | | | | |
| 1 | **CCDC82**: coiled-coil domain containing 82 | NM_024725 | HS | 86 |
| 1 | **OTUD4**: OTU domain containing 4 | XR_084597 | BT | 83 |
| 1 | **REEP5**: receptor accessory protein 5 | NM_001046605 | BT | 75 |
| 1 | **ST13:** suppression of tumorigenicity 13 (colon carcinoma) (Hsp70 interacting protein) | BC052982 | HS | 84 |
| 1 | **WAC**: WW domain-containing adapter with a coiled-coil | XM_611722 | BT | 94 |
| **Free radical metabolism** | | | | |
| 1 | **CYB561D2**: cytochrome b-561 domain containing 2 | NM_007022 | HS | 79 |
| 1 | **MDH1**: malate dehydrogenase 1, NAD (soluble) | NM_001009329 | FC | 93 |
| 1 | **NHLRC2**: NHL repeat containing 2 | XM_508046 | PT | 95 |
| 1 | **NDUFB8**: NADH dehydrogenase (ubiquinone) 1 beta subcomplex 8 19kDa | XM_001500281 | EC | 99 |
| 1 | **TCP1**: t-complex 1 | XM_001500650 | EC | 99 |
| **DNA/RNA associated proteins** | | | | |
| 1 | **BZW1**: Basic leucine zipper and W2 domains 1 | XM_001503602 | EC | 99 |
| 1 | **CSTF1**: cleavage stimulation factor, 3 pre-RNA subunit 1 | XM_001489360 | EC | 99 |
| 3 | **EEF1A1:** eukaryotic translation elongation factor 1 alpha 1 | NM_001081781 | EC | 99 |
| 1 | **EIF3D**: eukaryotic translation initiation factor 3 subunit D | XM_001500445 | EC | 100 |
| 1 | **EIF3E**: eukaryotic translation initiation factor 3 subunit E | BT025478 | BT | 94 |
| 1 | **GFM1**: elongation factor, mitochondrial 1 | BT025478 | HS | 71 |
| 1 | **HNRNPA0:** heterogeneous nuclear ribonucleoprotein A0 | XM_593096 | BT | 90 |
| 2 | **HNRNPA3**: heterogeneous nuclear ribonucleoprotein A3 | NM_194247 | HS | 97 |
| 1 | **HNRPDL**: heterogeneous nuclear ribonucleoprotein D-like | AY246721 | EC | 99 |
| 1 | **HNRNPH2**: heterogeneous nuclear ribonucleoprotein H2 (H') | XM_001493778 | EC | 99 |
| 2 | **LARP4**: La ribonucleoprotein domain family member 4 | XM_001504251 | EC | 100 |
| 1 | **LARP5**: La ribonucleoprotein domain family member 5 | BC152443 | HS | 87 |
| 1 | **MTRF1L**: Mitochondrial translational release factor 1-like | XM_001501586 | EC | 99 |
| 1 | **RPL5**: ribosomal protein L5 | NM_001081885 | EC | 100 |
| 1 | **RPS18**: ribosomal protein S18 | XM_001497064 | EC | 100 |
| 1 | **RPS28**: ribosomal protein S28 | XM_001504640 | EC | 99 |
| 1 | **SNRPC**: small nuclear ribonucleoprotein polypeptide C | XM_001498428 | EC | 99 |
| 1 | **SRP72**: signal recognition particle 72kDa | XM_002918109 | AM | 82 |
| 1 | **XRCC2**: X-ray repair complementing defective repair in Chinese hamster cells 2 | XM_00149740 | EC | 100 |
| 2 | **DDX60:** DEAD (Asp-Glu-Ala-Asp) box polypeptide 60 | XM_001082147 | MM | 78 |
| 1 | **IWS1:** IWS1 homolog | XM_001915606 | EC | 99 |
| 1 | **REXO2:** RNA exonuclease 2 homolog | XM_001502187 | EC | 99 |
| 2 | **RPL21:** ribosomal protein L21 | XM_001491519 | EC | 99 |
| 1 | **RPS6:** ribosomal protein S6 | XM_001496363 | EC | 99 |
| **Gene transcription** | | | | |
| 1 | **BAZ2B**: bromodomain adjacent to zinc finger domain 2B | BC012576 | HS | 94 |
| 1 | **CNOT7**: CCR4-NOT transcription complex subunit 7 | XM_001488465 | EC | 98 |
| 1 | **HBP1**: HMG-box transcription factor 1 | XM_001490734 | EC | 99 |
| 1 | **ZNF292**: Zinc finger protein 292 | XM_539029 | CF | 91 |
| **Metal ion binding** | | | | |
| 1 | **RNF170**: ring finger protein 170 | XM_003311708 | PT | 79 |
| 1 | **ZFYVE26:** zinc finger, FYVE domain containing 26 | XM_592247 | BT | 85 |
| **Sequences coding with uncharacterize sequences - Group II** | | | | |
| 1 | **GMPS:** guanine monophosphate synthetase | XM_001488228 | EC | 99 |
| 7 | Naqu mitochondrion | EF597513 | EC | 100 |
| 1 | **CCDC93:** coiled-coil domain containing 93 | XM_001488114 | EC | 97 |
| 1 | **LYRM7**: Lyrm7 homolog (mouse) | NM_181705 | HS | 80 |
| 1 | BAC clone CH251-347C9 | AC159217 | PT | 74 |
| 1 | DNA sequence from clone RP6-11O7 | AL031589 | HS | 73 |
| 1 | uncharacterized protein FLJ36031-like | XM_002818333 | PA | 81 |
| 1 | DNA sequence from clone RP11-520H16 | AL359753 | HS | 74 |
| 1 | clone XX-265A5 | AC186686 | CF | 79 |
| 1 | similar to KIAA2007 protein | XM_001492338 | EC | 98 |
| 1 | chromosome 1 open reading frame 141 (C1orf141) | NM_001013674 | HS | 77 |
| 1 | clone CH241-280K8 | AC124156 | EC | 99 |
| 1 | cDNA DKFZp459N1711 | CR861340 | PP | 78 |
| 2 | hypothetical protein LOC100053089 | XM_001502380 | EC | 100 |
| 1 | chromosome 8 open reading frame 4 (cDNA clone MGC:128767 IMAGE:7988712) | BC103283 | BT | 78 |
| 1 | clone RP11-539E19 | AC069079 | HS | 75 |
| 1 | BAC clone GS1-345D13 from 7 | AC002076 | HS | 74 |
| 1 | DNA sequence from clone RP11-624L12 | AL365364 | HS | 78 |
| 1 | chromosome 14 DNA sequence BAC R-896J10 of library RPCI-11 | AL132857 | HS | 74 |
| 1 | BAC clone CH250-378K15 | AC197276 | MM | 70 |
| 1 | cDNA FLJ13585 fis, clone PLACE1009150 | AK023647 | HS | 80 |
| 1 | chromosome X, clone XX-146G22 | AC186068 | CF | 76 |
| 1 | clone:AMP010011G04, expressed in alveolar macrophage | AK230581 | SC | 79 |
| 1 | DNA sequence from clone RP11-24J23 | AL445205 | HS | 81 |
| 1 | BAC clone CH242-347J6 | CU606901 | SC | 84 |
| 1 | chromosome 3 open reading frame 64 | BC028935 | HS | 78 |
| 1 | similar to hCG2043237 | XM_001493651 | EC | 90 |
| 1 | clone:LNG010075G10, expressed in lung | AK232078 | SC | 78 |
| 1 | cDNA FLJ13585 fis, clone PLACE1009150 | AK023647 | HS | 79 |
| 1 | BAC clone CH271-148E8 | AC198152 | NL | 87 |
| 2 | similar to hCG2043240 (LOC100072855) | XM_001502913 | EC | 85 |
| 1 | cDNA DKFZp586F1322 (from clone DKFZp586F1322) | AL050172 | HS | 95 |
| 1 | BAC CH250-204L23 | AC198166 | RM | 83 |
| 1 | clone 2H576 | HM175907 | EC | 82 |
| 1 | CT020027B20G06 Equine Articular Cartilage cDNA Library ( cDNA clone CT020027B20G06) | CX600828 | EC | 100 |
| 1 | CT02034A2E06 Equine Articular Cartilage cDNA Library (cDNA clone CT02034A2E06) | CX603018 | EC | 96 |
| 1 | HL01021B1E02 Equine lymphocyte cDNA Library (cDNA clone HL01021B1E02) | DN507572 | EC | 98 |
| 1 | 1514334 MARC 3PIG | DY410347 | SC | 76 |
| 1 | rese39c_e2.y1 ese | EW147235 | SC | 77 |
| 1 | CT02031A1G03 Equine Articular Cartilage cDNA Library (cDNA clone CT02031A1G03) | CX601918 | EC | 100 |
| 1 | Genomic chr5 |  |  |  |
| 1 | Genomic chr10 |  |  |  |
| 1 | Genomic chr25 |  |  |  |
| 2 | Genomic chrUn |  |  |  |
| 1 | Genomic chr27 |  |  |  |
| 1 | Genomic chrUn |  |  |  |
| 1 | Genomic chr2 |  |  |  |
| 1 | Genomic chr13 |  |  |  |

Fr (frequency) indicates the redundancy for each cDNA clone. Genes name used are recommended by the HUGO Gene Nomenclature Committee (HGNC) when applicable. SC, *Sus crofa*; MM, *Macaca mulatta*; EC, *Equus caballus*; BT, *Bos taurus*; PT, *Pan troglodytes*; OA, *Ovis aries*; HS, *Homo sapiens*; CF, *Canis familiaris*; FC, *Felis catus*; MF, *Macaca fascicularis*; NL, *Nomascus leucogenys*; RM, *Rhesus macaque*; AM, *Ailuropoda melanoleuca*; PA, *Pongo abelii*; TT, *Tursiops truncatus*; PP, *Pongo pygmaeus*; BAC, bacterial artificial chromosome.
